# The Proportion of Normalized Hips with Growth in Japanese Adolescents Aged > 10 years with Acetabular Dysplasia who Presented with Suspected Scoliosis

**DOI:** 10.1007/s43465-023-01065-4

**Published:** 2023-12-08

**Authors:** Takahiro Nishimura, Hideaki Watanabe, Naoya Taki, Ichiro Kikkawa

**Affiliations:** 1 Department of Orthopedic Surgery, Jichi Medical University, 3311-1 Yakushiji, Shimotsuke, Tochigi, 329-0498 Japan; 2 Department of Pediatric Orthopedics and Orthopedic Surgery, Jichi Children’s Medical Center, 3311-1 Yakushiji, Shimotsuke, Tochigi, 329-0498 Japan; 3 Department of Orthopedic Surgery, Nasu Central Hospital, 1453 Shimoishigami, Otawara, Tochigi, 324-0036 Japan

**Keywords:** Acetabular dysplasia, Adolescence, Normalization, Japanese

## Abstract

**Background:**

If asymptomatic acetabular dysplasia (AD) is incidentally identified in adolescence, it is difficult to determine the appropriate follow-up or treatment strategy because the acetabulum is still developing. We investigated the rate of AD normalization at the end of acetabular growth.

**Methods:**

This cross-sectional study involved 653 patients (1306 hips) aged 10–14 years with scoliosis or suspected scoliosis. All patients underwent plain standing whole-spine radiography (with the pelvis included) at the first visit. We measured the lateral center–edge angle, Sharp angle, Tönnis angle, and acetabular head index on radiographs. The criterion for AD was a lateral center–edge angle of < 20°. We extracted the data of patients aged < 12 (10–11) years and ≥ 12 (12–14) years with AD. Furthermore, we analyzed the radiographic follow-up data at 15 years of age to identify the AD normalization rate.

**Results:**

AD was diagnosed in 19 hips from patients aged < 12 years and in 36 hips from patients aged ≥ 12 years. The AD normalization rate at 15 years of age was 31.6% in those diagnosed at < 12 years of age and 5.6% in those diagnosed at ≥ 12 years of age.

**Conclusion:**

AD in adolescence was predictive of AD at the end of growth in 95% of cases diagnosed at ≥ 12 years of age compared with approximately 70% of cases diagnosed at < 12 years of age. Surgical treatment before completion of acetabular growth is beneficial for acetabular remodeling, but the decision to operate should be carefully evaluated in patients aged < 12 years.

**Graphical Abstract:**

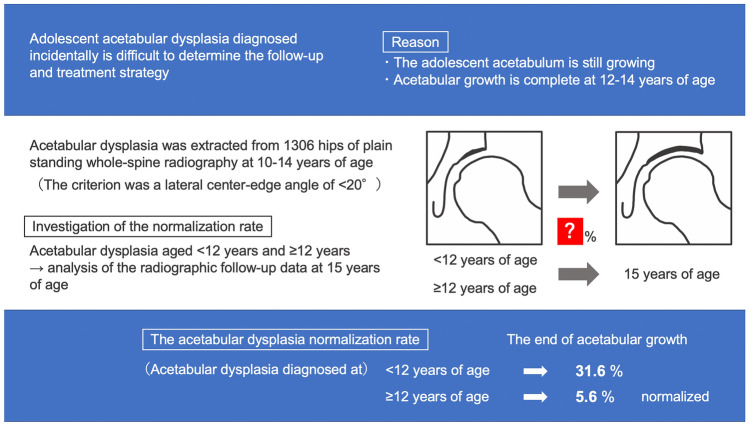

**Supplementary Information:**

The online version contains supplementary material available at 10.1007/s43465-023-01065-4.

## Introduction

In adolescents, acetabular dysplasia (AD) is defined according to several criteria, including the lateral center–edge angle (LCEA), with an LCEA of < 20° regardless of age, an LCEA of < 15° in people aged < 15 years or an LCEA of < 10° in people aged < 14 years, and an LCEA of < 15° in people aged > 14 years [1–3]. It is better to perform early corrective surgery if AD is symptomatic, there is residual subluxation, and the LCEA is < 10°–20°. However, there is no definite opinion on this matter [4, 5]. When asymptomatic AD without a history of dislocation is incidentally identified in adolescence, it may be difficult to determine the appropriate follow-up or treatment strategy.

Acetabular growth is concluded with closure of the triradiate cartilage and secondary ossification centers of the acetabulum, which occurs at around the age of 12–14 years [6, 7]. Kobayashi et al. investigated the increase in the LCEA between the age of 3 and 18 years, demonstrating that 93.4% of the final normal acetabulum had normalized at 12 years of age, but if AD persisted at 12 years of age, it did not normalize thereafter [8]. Loder et al. reported that surgery in patients aged < 14 years can help to achieve remodeling after osteotomy [9].

In this study, we investigated the rate of asymptomatic AD normalization with age in people in whom AD was incidentally identified after the age of 10 years. We also calculated the predicted probability of AD at the end of acetabular growth in people aged < 12 years and ≥ 12 years at the first AD diagnosis.

## Materials and Methods

**Fig. 1:**
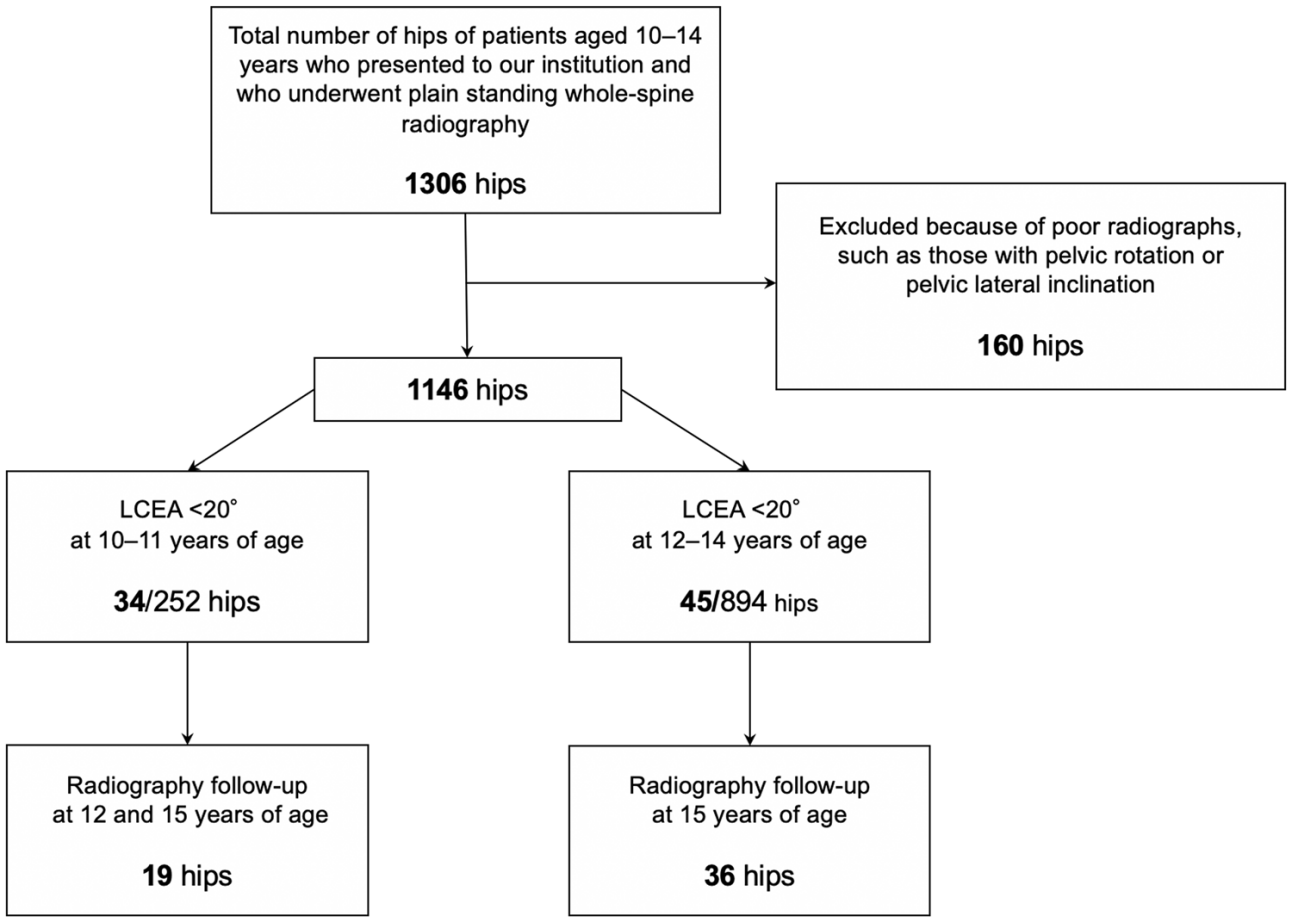
Patient flow and data extraction. LCEA, lateral center-edge angle

The study protocol was approved by the ethics review board of our university. Informed consent was obtained from all persons. This cross-sectional study included people who visited our outpatient department owing to scoliosis or suspected scoliosis from February 2006 to March 2020. A total of 653 patients (1306 hips) aged 10–14 years were included in the study. All patients underwent plain standing whole-spine (including the pelvis) radiography at the first visit. All patients’ hip joints were asymptomatic. No patients had a history of or treatment for developmental dysplasia of the hip in childhood. Eighty patients (160 hips) in whom measurements of acetabular radiographic parameters could not be properly obtained because of pelvic rotation or pelvic lateral inclination were excluded. In total, 1146 hips were measured in 573 patients. AD was defined as an LCEA of < 20°, and all the acetabulae were measured on the initial radiographs. Then, we retrospectively selected incidentally identified cases of AD in patients aged < 12 (10–11) years and ≥ 12 (12–14) years, divided by age. Furthermore, among the patients with AD, we extracted the radiographic follow-up data at 12 and 15 years of age for patients diagnosed at < 12 years of age, and the radiographic follow-up data at 15 years of age for patients diagnosed at ≥ 12 years of age. In other words, the same patients with AD aged < 12 years and ≥ 12 years were followed to 15 years of age (Fig. 1).

The radiographs were obtained digitally. The measured parameters were the LCEA [1], Sharp angle [10], Tönnis angle [11], and acetabular head index (AHI) [12]. All measurements were made by one orthopedic specialist surgeon (first author).

The primary outcome was the rate of AD normalization (defined as an LCEA of ≥ 20°) with aging (measured at 15 years of age) in patients with incidentally identified AD (identified at < 12 years or ≥ 12 years of age). We divided the patients into the final AD group and the normalized group according to the final LCEA at 15 years of age (secondary outcome), and we compared the LCEA, Sharp angle, Tönnis angle, and AHI between the two groups.

### Statistical Analysis

The statistical analysis was performed using SPSS, version 25 (IBM Corp., Armonk, NY, USA). The Wilcoxon signed rank test was used to compare the parameters at different time points for each patient, while the Mann–Whitney U-test was used to compare the data between the two groups. P values ≤ 0.05 were considered statistically significant.

## Results

AD was present in 19 of 252 hips in patients aged < 12 (10–11) years, namely 8 hips (3 boys and 5 girls) in patients aged 10 years and 11 hips (4 boys and 7 girls) in patients aged 11 years. AD was present in 36 of 894 hips in patients aged ≥ 12 (12–14) years, namely 14 hips in patients aged 12 years, 8 hips in patients aged 13 years, and 14 hips in patients aged 14 years. All cases of AD in patients aged ≥ 12 (12–14) years were girls.

### AD Normalization Rate with Aging

**Fig. 2:**
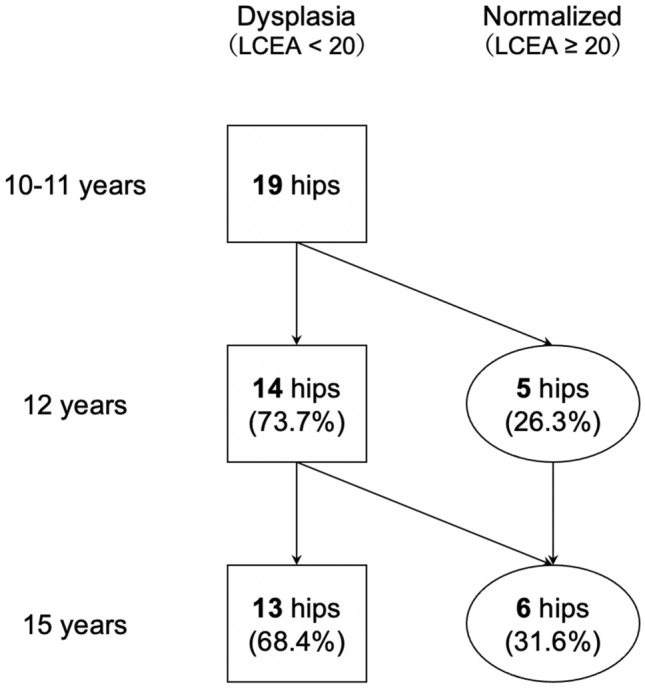
Acetabular growth over time in patients with AD at 10–11 years of age (n = 19). AD, acetabular dysplasia; LCEA, lateral center-edge angle

**Fig. 3:**
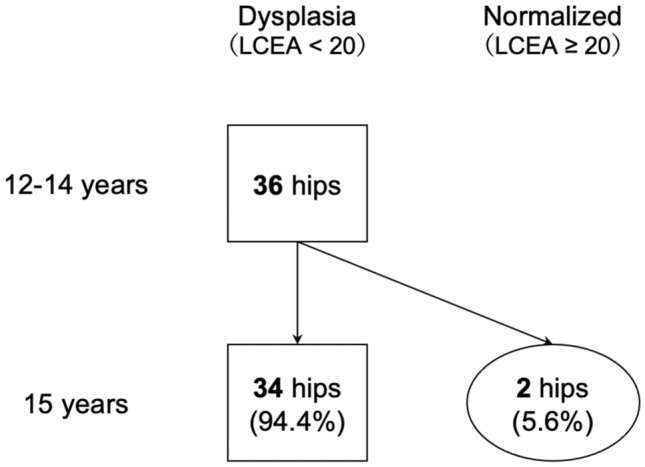
Acetabular growth over time in patients with AD at 12–14 years of age (n = 36). AD, acetabular dysplasia; LCEA, lateral center-edge angle

The rate of AD normalization with aging in patients diagnosed with AD at < 12 years of age was 26.3% (5 hips) at 12 years of age and 31.6% (6 hips) at 15 years of age (Fig. 2). The rate of AD normalization with aging in those diagnosed with AD at ≥ 12 years of age was 5.6% (2 hips) at 15 years of age (Fig. 3).

### Comparison Between the Final Dysplasia and Normalized Groups

We divided the patients into two groups according to whether the final LCEA at 15 years of age was illustrative of dysplasia or normalization.

For AD diagnosed in patients aged < 12 years, the AD group included 13 hips, while the normalized group included 6 hips. The comparison of radiographic parameters (LCEA, Sharp angle, Tönnis angle, and AHI) with aging between the AD and normalized groups is summarized in Table 1. The LCEA (± standard deviation) was 16.3° ± 2.2° in the AD group and 17.3° ± 1.5° in the normalized group at 10–11 years of age, 17.5° ± 2.2° in the AD group and 20.8° ± 1.9° in the normalized group at 12 years of age, and 17.7° ± 1.4° in the AD group and 22.7° ± 2.4° in the normalized group at 15 years of age. For each parameter, the final values indicated acetabular growth in both groups. There was a significant difference in the final LCEA and Tönnis angle (P = 0.01). There was no significant difference between the two groups in any parameter at 10–11 years of age.

**Table 1:** Radiographic parameter comparison between the final dysplasia group (D, n = 13) and the normalized group (N, n = 6) in patients with acetabular dysplasia diagnosed at < 12 years of age (n = 19)

	10–11 Years			12 Years			15 Years		
	D	N	p-value	D	N	p-value	D	N	p-value
LCEA (°)	16.3 ± 2.2	17.3 ± 1.5	0.32	17.5 ± 2.2	20.8 ± 1.9	0.01	17.7 ± 1.4	22.7 ± 2.4	0.01
Sharp angle (°)	50.5 ± 3.2	50.0 ± 1.3	0.74	50.2 ± 1.9	49.0 ± 0.9	0.15	49.8 ± 2.2	47.8 ± 1.9	0.08
Tönnis angle (°)	12.2 ± 1.5	13.0 ± 1.9	0.30	10.9 ± 1.4	10.3 ± 1.6	0.43	10.8 ± 1.6	8.2 ± 2.0	0.01
AHI (%)	71.2 ± 4.7	74.2 ± 3.9	0.19	73.6 ± 5.1	76.7 ± 3.4	0.20	74.0 ± 5.0	78.5 ± 3.2	0.06

Results are presented as mean ± standard deviation

*LCEA* lateral center-edge angle, *AHI* acetabular head index

For AD diagnosed in patients aged ≥ 12 years, the AD group included 34 hips and the normalized group included 2 hips. The comparison of radiographic parameters with aging between the AD and normalized groups is summarized in Table 2. The LCEA was 17.2° ± 1.5° in the AD group and 18.0° ± 0.0° in the normalized group at 12–14 years of age, and 17.1° ± 1.5° in the AD group and 21.5° ± 0.7° in the normalized group at 15 years of age. There was a significant difference between the two groups in the final LCEA (P = 0.01) and Tönnis angle (P = 0.04). However, there was no significant difference between the two groups in any parameter at 12–14 years of age.

**Table 2:** Radiographic parameter comparison between the final dysplasia group (D, n = 34) and the normalized group (N, n = 2) in acetabular dysplasia patients diagnosed at over 12 years of age (n = 36)

	12–14 Years			15 Years		
	D	N	p-value	D	N	p-value
LCEA (°)	17.2 ± 1.5	18.0 ± 0.0	0.42	17.1 ± 1.5	21.5 ± 0.7	0.01
Sharp angle (°)	47.8 ± 2.7	49.5 ± 2.1	0.38	49.1 ± 2.1	46.5 ± 2.1	0.10
Tönnis angle (°)	11.3 ± 2.4	11.0 ± 0.0	0.88	11.0 ± 2.2	7.5 ± 0.7	0.04
AHI (%)	73.9 ± 3.6	74.5 ± 2.1	0.83	73.8 ± 3.3	77.5 ± 0.7	0.13

Results are presented as mean ± standard deviation

*LCEA* lateral center-edge angle, *AHI* acetabular head index

## Discussion

The goal of surgical treatment for adolescent AD is to avoid future early-onset osteoarthritis and total hip arthroplasty (THA). AD has long been implicated as a cause of osteoarthritis [13]. For example, Cooperman et al. performed a 22-year follow-up of hips with an LCEA of < 20° and reported that all patients had developed osteoarthritis by the age of 65 years [14]. Murphy et al. reported that osteoarthritis progresses in patients with an LCEA of < 16° [4]. Moreover, Okano et al. reported that 65% of patients undergoing THA for osteoarthritis had AD without a history of developmental dislocation of the hip [15]. Clohisy et al. reported that osteoarthritis in 48.4% of patients aged < 50 years who underwent THA was attributed to AD [16]. In the study of Wyles et al., AD was the greatest risk factor for progression of osteoarthritis and THA (hazard ratio, 5.0). One third of patients with AD underwent THA within 10 years, and two-thirds of patients with AD developed osteoarthritis within 20 years [17]. Even though AD is known to cause osteoarthritis and lead to THA, the diagnostic criteria and treatment strategies for AD in adolescents have not been firmly established. Even the LCEA, which is the most commonly used angle for evaluation, is inconsistently used in the diagnostic criteria for AD [1–3].

Acetabular growth is one of the factors that make it difficult to determine the treatment strategy for AD in adolescents. The end of acetabular growth and closure of the triradiate cartilage and secondary ossification centers of the acetabulum occur at around the age of 12–14 years [6, 7]. Than et al. reported that the LCEA gradually increases from 10 to 15 years of age and remains almost unchanged after 15 years of age [18]. Kobayashi et al. investigated the contralateral side of AD from 3 to 18 years of age and observed an improvement in the LCEA between 9 and 12 years of age. The final normalized group demonstrated that 71.1% of cases had normalized at 9 years of age, 93.4% had normalized at 12 years of age, and 98.7% had normalized at 15 years of age. Conversely, if AD persisted at the age of 12 years, it never normalized thereafter [8]. Noritake et al. reported that acetabular growth at 8–12 years of age normally develops from the secondary ossification center at the acetabular rim, but the secondary ossification center is lacking or insufficient in patients with AD, leading to primary AD [19]. The present study showed that 31.6% of hips with AD diagnosed in patients aged < 12 years and 5.6% of hips with AD diagnosed in patients aged ≥ 12 years had normalized by 15 years of age. This result was considered to support Kobayashi et al.’s findings [8]. Based on our results, we estimated the predicted probability of future AD at 15 years of age based on the age at which AD was initially diagnosed, although this is only a rough index. The predicted probability was approximately 70% in those diagnosed with AD at < 12 years of age and 95% in those diagnosed at ≥ 12 years of age (Table 3). It is thought that acetabular growth is complete and the morphology is stable at the age of 12 years, so it is necessary to decide carefully whether surgical treatment is suitable. In the subanalysis, the normalized group and the final AD group were compared to evaluate differences in parameters other than the LCEA. There were significant differences in the LCEA and Tönnis angle at 15 years of age. Kobayashi et al. reported that, similar to the LCEA, there was a difference in the Sharp angle between the normalized group and the AD group after 12 years of age [8].

**Table 3:** Predicted probability of future acetabular dysplasia (15 years of age) at the age acetabular dysplasia was identified

Age	Predicted probability
< 12 Years	70%
≥ 12 Years	95%

Surgical treatment for AD, especially periacetabular osteotomy (PAO), can provide good outcomes with low complication rates. The mid- and long-term survival rate after PAO has been reported as 88% at 10 years, 61% at 20 years, and 29% at 30 years [20]. Other studies reported survival rates of 73% at 20 years [21]. The major complication rate of PAO was 5.9%–7.0% in previous studies [22, 23]. However, these reports included patients aged > 20 years. Loder et al. reported that performing PAO (in this report, triple osteotomy) in patients aged < 14 years was advantageous for postoperative remodeling, and it was also reported that remodeling affects the size of the birth canal, reducing the risk of caesarean section in women [9]. Hingsammer et al. suggested that PAO changes the mechanical loading of the articular cartilage in the acetabulum, which in turn normalizes the cartilage matrix composition [24]. Therefore, PAO for AD in adolescents can be expected to favorably affect acetabular remodeling, including pelvic ring remodeling and cartilage normalization, if it is performed before acetabular growth is complete. However, based on the results of our study, the predicted probability of future AD in patients diagnosed with AD at < 12 years of age was approximately 70%, so the fact that the acetabulum may still be growing in younger adolescents should be considered.

This study has several limitations that should be noted. First, all of the patients had scoliosis or suspected scoliosis, were aged 10–15 years, and were Japanese, which may limit the generalizability of the findings. Moreover, the data were analyzed by chronological age only, without consideration of individual differences in skeletal maturation and sex. For example, we quantified remodeling of the acetabulum to the end of its growth, using 12 years as the reference age; however, sex differences may have existed among the patients. Regarding sex differences, both the fact that the study population comprised patients who presented with suspected scoliosis and the purpose of the study was related to AD may have led to sex bias. Both the incidence of adolescent idiopathic scoliosis (AIS) (732.3 vs. 338.8/100,000 person-years) and AD (2.07% vs. 0.75%) are higher in female patients compared with male patients, respectively [25, 26]. Additionally, the fact that this study included patients with scoliosis and suspected scoliosis must also be mentioned, as the pelvis may differ from a normal pelvis in people with scoliosis. An association in development between scoliosis and acetabular dysplasia in adolescents has not been reported previously. However, radiographic differences between the pelvis of scoliosis patients and the normal pelvis have been reported. Banno et al. reported that pelvic obliquity was present in 23% of AIS patients with coronal alignment [27]. Wang et al. reported that the majority of AIS patients with a right major thoracic or major left thoracolumbar/lumbar curve have pelvic axial rotation in the transverse plane [28]. Karam et al. reported three-dimensional deformity of the acetabulum with AIS, as follows: The acetabulum was more abducted at the lower side vs upper side, the acetabulum was anteverted, and there was a lack of anterior coverage. These alterations were related to pelvic obliquity [29]. Therefore, to reduce the effect of pelvic obliquity as much as possible, the radiographic methods were standardized, and cases unsuitable for measurement of acetabular parameters because of pelvic rotation or pelvic lateral inclination were excluded. Regarding the radiographic method, when plain standing whole-spine radiography was performed in our hospital, the patients stood with their patellae forward and locked and their feet shoulder-width apart, and they looked straight ahead with their elbows bent and made a fist with each hand in the bilateral supraclavicular fossae. Furthermore, the acetabular radiographic parameters measured in this study were compared between patients with a Cobb angle < 10° and those with a Cobb angle ≥ 10°, and no significant differences were found between patients with and without scoliosis (Supplemental file). However, we consider that the results of this study cannot be generalized because all the subjects had scoliosis or suspected scoliosis. Second, the radiographs were obtained in the anteroposterior whole-spine view, which differed from a normal standing anteroposterior pelvic radiograph in the irradiation distance and the center of the X-ray beam. Furthermore, the center of focus changed slightly depending on the growth in height. However, we reported previously that there is no significant effect of height of body on the problems of using whole-spine radiographs for evaluating the acetabular parameters [30]. Regarding the fact that one investigator performed the measurements, in the same previous study, we reported intra-rater reliability of more than moderate for LCEA, Sharp angle, Tönnis angle, and AHI [30]. Finally, the cohort size was very small and regional, in the present study. There were geographic limitations and problems of applicability of a Japanese-only dataset. Additionally, radiographs in patients younger than 10 years and older than 15 years could not be evaluated. No conclusions could be drawn other than that acetabular remodeling after the age of 12 years in patients with AD is rare. In particular, AD normalized with aging in only two hips in the group aged ≥ 12 years. The number was so small that it was unclear whether the result was statistically relevant. We believe that studies with higher numbers of patients and longer-term follow-up from a younger age compared with our study are required to examine age-related normalization of AD in adolescents.

In summary, AD in adolescence was predictive of AD at the end of growth in 95% of cases diagnosed at ≥ 12 years of age compared with approximately 70% of cases diagnosed at < 12 years of age. Surgical treatment for AD in adolescents is beneficial for acetabular remodeling if it can be performed before completion of acetabular growth, but at < 12 years of age, the decision to perform surgery should be made with caution. The results of this study may help to determine appropriate follow-up and treatment strategies for AD in adolescents.

### Supplementary Information

Below is the link to the electronic supplementary material.

Supplementary file1 (DOCX 15 KB)

## Data Availability

The datasets used and/or analyzed during the current study are available from the corresponding author on reasonable request.
