# Supplementary material for: The Proportion of Normalized Hips with Growth in Japanese Adolescents Aged > 10 years with Acetabular Dysplasia who Presented with Suspected Scoliosis
Source: Indian J Orthop. 2023 Dec 8;58(2):169–75. doi: 10.1007/s43465-023-01065-4 (PMC10830976; doi:10.1007/s43465-023-01065-4)
Supplement: Supplementary file 1 — Supplementary file1 (DOCX 15 KB) [file 43465_2023_1065_MOESM1_ESM.docx]

**Supplement file**

Radiographic parameters comparison between patients with a Cobb angle <10° and those with a Cobb angle ≥10°

|  | Cobb angle <10° (n=294) | Cobb angle ≥10° (n=852) | p |
| --- | --- | --- | --- |
| Cobb angle (°) | 5.4 ±2.9 | 22.8 ±9.9 | 0.01 |
| L4 coronal tilt (°) | 2.3 ±1.9 | 7.2 ±4.7 | 0.01 |
| LCEA (°) | 27.6 ±5.4 | 27.8 ±4.8 | 0.38 |
| Sharp angle (°) | 44.1 ±2.8 | 44.1 ±3.2 | 0.73 |
| Tönnis angle (°) | 5.1 ±4.8 | 5.1 ±3.6 | 0.90 |
| AHI (%) | 82.2 ±6.0 | 82.0 ±5.4 | 0.58 |

The mean and standard deviation

LCEA, lateral center-edge angle; AHI, acetabular head index
